# Supplementary material for: Traditional lifestyle factors partly mediate the association of socioeconomic position with intrahepatic lipid content: The Maastricht study
Source: JHEP Rep. 2023 Jul 18;5(11):100855. doi: 10.1016/j.jhepr.2023.100855 (PMC10522893; doi:10.1016/j.jhepr.2023.100855)
Supplement: Multimedia component 1 [file mmc1.pdf]

Traditional lifestyle factors partly mediate the association of socioeconomic position with intrahepatic lipid content: The Maastricht study

Zhewen Ren, Hans Bosma, Anke Wesselius, Simone JPM Eussen, M. Eline Kooi, Carla J.H. van der Kallen, Annemarie Koster, Marleen van Greevenbroek, Pieter Dagnelie, Coen DA Stehouwer, Martijn CGJ Brouwers

Table of contents

Supplementary figures..2

Supplementary tables..separate excel file

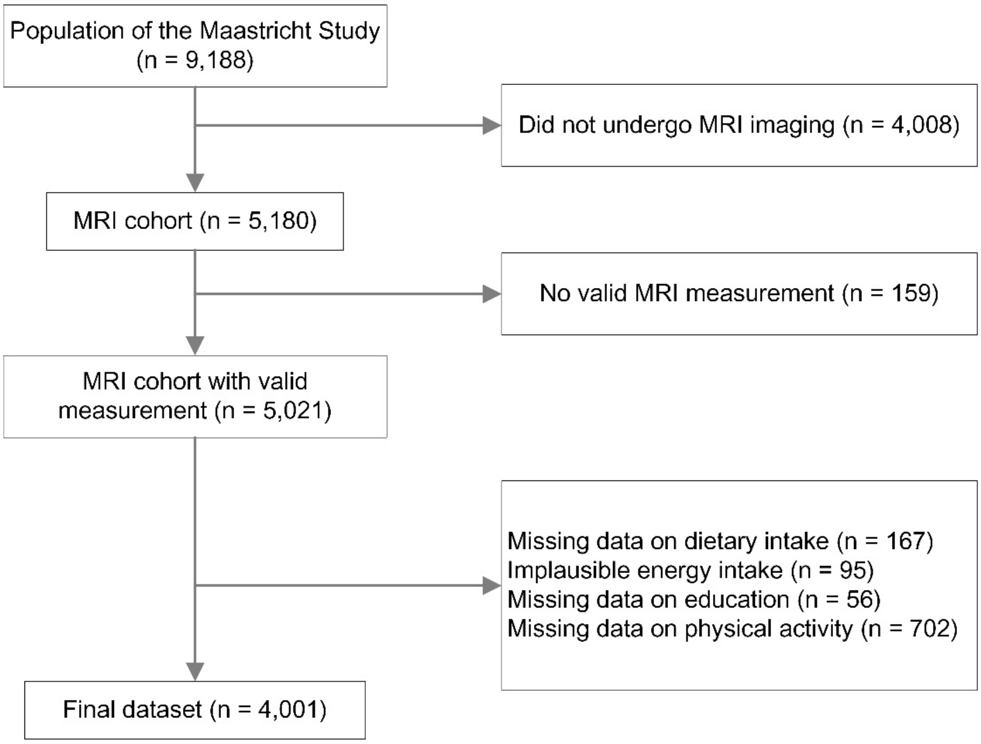

Fig. S1. Flowchart of the education dataset.

Abbreviation: MRI: Magnetic resonance imaging.

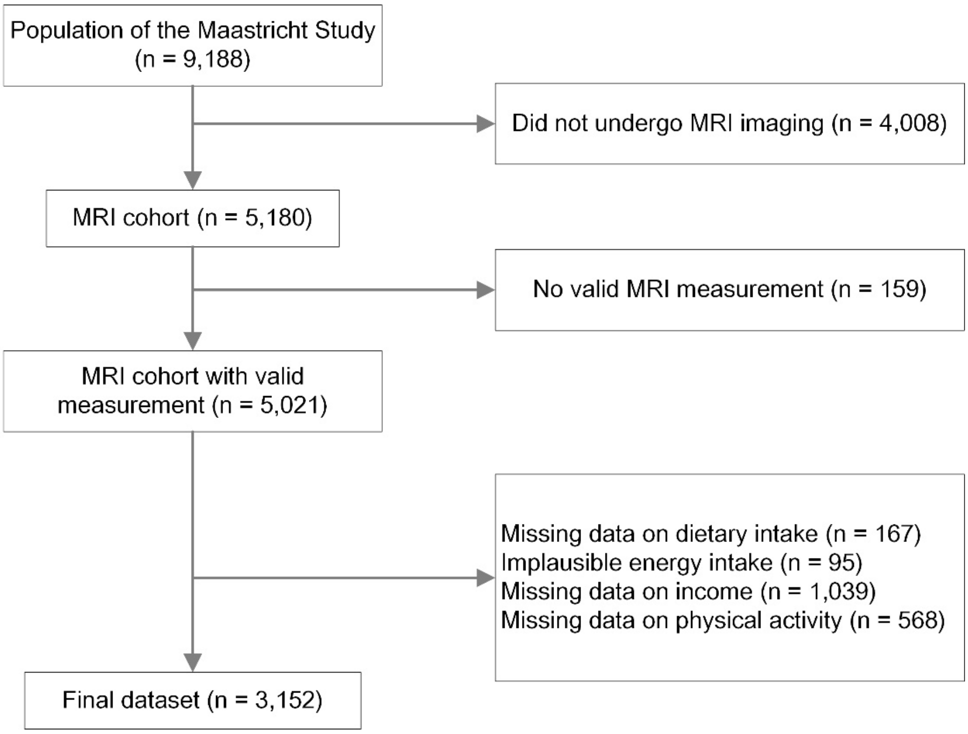

Fig. S2. Flowchart of the income dataset.

Abbreviation: MRI: Magnetic resonance imaging.

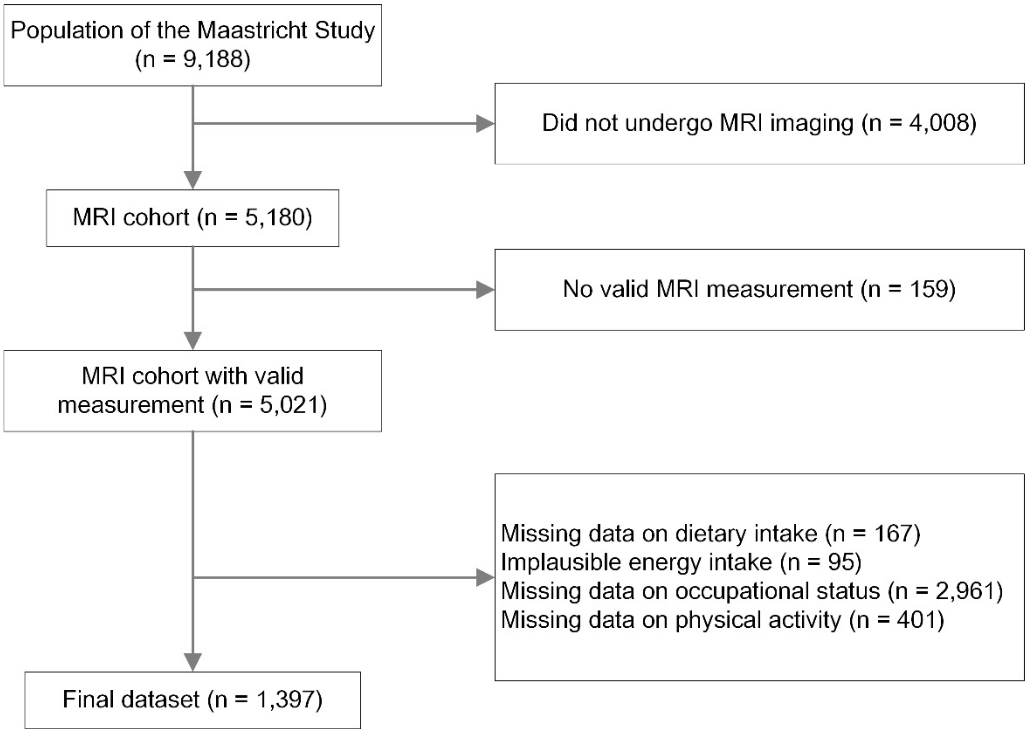

Fig. S3. Flowchart of the occupation dataset.

Abbreviation: MRI: Magnetic resonance imaging.

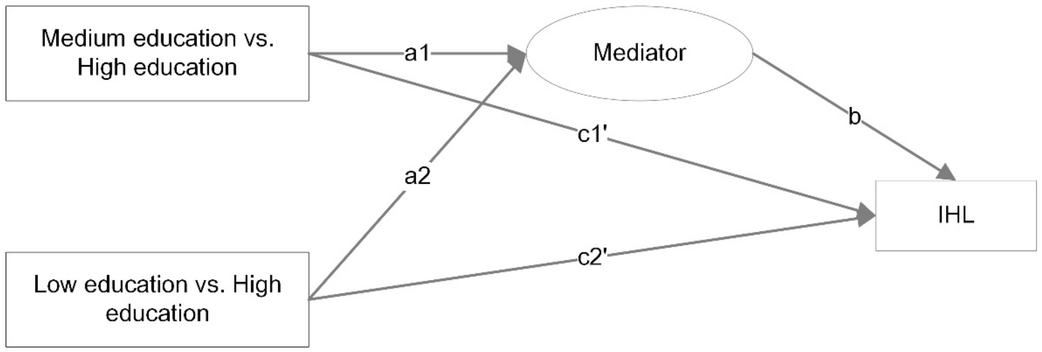

Fig. S4. **Multiple parallel mediation model to assess the mediation effects of lifestyle factors on the relationship between education and IHL content.**

a1*b and a2*b represent the indirect effects, whereas c1', c2' represent the direct effects. Abbreviations: IHL: intrahepatic lipid content.
