# Supplementary material for: Traditional lifestyle factors partly mediate the association of socioeconomic position with intrahepatic lipid content: The Maastricht study
Source: JHEP Rep. 2023 Jul 18;5(11):100855. doi: 10.1016/j.jhepr.2023.100855 (PMC10522893; doi:10.1016/j.jhepr.2023.100855)
Supplement: Multimedia component 5 [file mmc5.pdf]

Traditional lifestyle factors partly mediate the association of socioeconomic position with intrahepatic lipid content: The Maastricht study

Zhewen Ren,[1,2,3] Hans Bosma,[4,5] Anke Wesselius,[6,7] Simone J.P.M. Eussen,[2,6,7] M. Eline Kooi,[2,8] Carla J.H. van der Kallen,[1,2] Annemarie Koster,[4,5] Marleen M.J. van Greevenbroek,[2,3] Pieter Dagnelie,[1,2] Coen D.A. Stehouwer,[1,2] Martijn C.G.J. Brouwers[1,2,5,*]

[1]Department of Internal Medicine, Maastricht University Medical Center, Maastricht, The Netherlands; [2]CARIM School for Cardiovascular Diseases, Maastricht University, Maastricht, The Netherlands; [3]Laboratory for Metabolism and Vascular Medicine, Maastricht University, Maastricht, The Netherlands; [4]Department of Social Medicine, Maastricht University, Maastricht, The Netherlands; [5]CAPHRI Care and Public Health Research Institute, Maastricht University, Maastricht, The Netherlands; [6]Department of Epidemiology, Maastricht University, Maastricht, The Netherlands; [7]NUTRIM School for Nutrition and Translational Research in Metabolism Maastricht University, Maastricht, The Netherlands; [8]Department of Radiology and Nuclear Medicine, Maastricht University Medical Center, Maastricht, The Netherlands

JHEP Reports **2023.** https://doi.org/10.1016/j.jhepr.2023.100855

Background & Aims: Recent studies have unveiled an association between socioeconomic position (SEP) and intrahepatic lipid (IHL) content. The aim of this study was to examine to what extent traditional lifestyle factors mediate the relationship between SEP and IHL content, independent of aetiology, and non-alcoholic fatty liver disease (NAFLD).
Methods: We used cross-sectional data derived from The Maastricht Study (N = 4,001; mean age: 60 years, 49% women, 32% low education level, 21% diabetes, 21% NAFLD). Education, income, and occupation were used as indicators of SEP. Physical activity (accelerometer), intake of total energy, alcohol, saturated fat, protein, vitamin E, dietary fibre, and fructose from sugar-sweetened beverages (SSBs) and fruit juice (food frequency questionnaires) were potential mediators. IHL content was quantified by magnetic resonance imaging. Age, sex, and type 2 diabetes were covariates. Multiple parallel mediation analyses (bootstraps = 10,000) were performed.
Results: Individuals with a low education level had a 1.056-fold higher IHL content (95% CI: 1.03–1.08) and a 44% greater NAFLD risk (OR:1.44; 95% CI:1.18–1.77) compared with those with higher education levels. Approximately 8.9% of educational disparity in risk of IHL content was attributable to moderate-to-vigorous physical activity; 6.3% to fructose intake from SSBs; 5.5% to dietary fibre; and -23% to alcohol. Approximately 8.7% of educational disparity in risk of NAFLD was attributable to moderate-to-vigorous physical activity; and 7.7% to fructose intake from SSBs. However, the indirect effect of these mediators was small (0.998 for IHL content and 1.045 for NAFLD) in comparison to the total effect. Similar results were found when income and occupation were used as SEP indicators.
Conclusions: Societal measures may alleviate the burden of NAFLD and further studies that identify mediators other than traditional lifestyle factors are warranted to define the relationship underlying SEP and IHL content.
Impact and implications: Individuals with a low or medium level of education, income, or occupational status had more fat accumulation in their livers than individuals with a higher education, income, or occupational status. This difference may be attributed to the influence of unhealthy lifestyle factors, such as reduced physical activity and a higher intake of sugar-sweetened beverages among individuals with lower socioeconomic position. Nevertheless, other yet unknown factors may also play a role.
© 2023 The Author(s). Published by Elsevier B.V. on behalf of European Association for the Study of the Liver (EASL). This is an open access article under the CC BY license (http://creativecommons.org/licenses/by/4.0/).

Introduction

The global prevalence of non-alcoholic fatty liver disease (NAFLD) has risen to pandemic proportions. Approximately 32.4% of adults, *i.e.* ~1.4 billion people, are affected by NAFLD worldwide.[1] These alarming numbers emphasise the need of effective preventive measures besides the identification of new pharmacological targets.

Many studies have identified lifestyle factors in the pathogenesis and progression of NAFLD. These factors include excess caloric intake,[2] high intake of fructose–in particular fructose from sugar-sweetened beverages (SSBs) and fruit juice[3,4]–and saturated fat,[5] low intake of protein and fibre,[2,6,7] vitamin E,[8] and low levels of physical activity.[9] Although excessive alcohol intake is by definition not a cause of NAFLD, some patients with fatty liver disease may have a dual aetiology, which not only involves

Keywords: Intrahepatic lipid content; Lifestyle; Mediation analysis; Non-alcoholic fatty liver disease; Socioeconomic.
Received 6 March 2023; received in revised form 2 June 2023; accepted 7 July 2023; available online 18 July 2023
* Corresponding author. Address: Department of Internal Medicine, Division of Endocrinology and Metabolic Diseases, Maastricht University Medical Center, PO Box 5800, 6202 AZ Maastricht, The Netherlands. Tel.: +31 433877019.
E-mail address: mcgj.brouwers@mumc.nl (M.C.G.J. Brouwers).

ELSEVIER
EASL The Home of Hepatology

the unhealthy lifestyle factors mentioned above but also includes a high alcohol intake.[10]

Interventions focusing on modifying lifestyle factors to treat and prevent the accumulation of IHL and the onset of NAFLD requires a better understanding of the factors that characterise an unhealthy lifestyle and how they relate with IHL content and NAFLD. Although many studies have examined the relationship between a low socioeconomic position (SEP) and an unhealthy lifestyle,[11–13] little is still known about the relationship between SEP and IHL content, and the potential mediating role of lifestyle factors therein.

Therefore, in the present study, we examined the relationship between three SEP indicators (educational level, income level, and occupational level) and IHL accumulation, independent of aetiology (as the primary outcome), and NAFLD (as a secondary analysis), and assessed to what extent these relationships were influenced by traditional lifestyle factors.

Patients and materials

This study was conducted in accordance with the Guideline for Reporting Mediation Analyses (AGReMA) statement,[14] see Table S1 for a checklist.

Study population

We used data from the Maastricht Study, a population-based cohort study enriched with type 2 diabetes patients (T2D).[15] In brief, this study focused on the aetiology, pathophysiology, complications, and comorbidities of T2D utilising an extensive phenotyping approach. All individuals aged between 40 and 75 years and living in the southern part of the Netherlands were eligible for participation.

The present study included cross-sectional data from 9,188 participants who completed baseline measurements from November 2010 until November 2020. Magnetic resonance imaging (MRI) measurements of the liver were performed from December 2013 onwards as part of the initial baseline workup and partly as a catch-up measurement (available in n = 5,180). As many individuals had missing data on income (n = 1,039) and occupational status (n = 2,961), we constructed three separate datasets for each SEP indicator, *i.e.* the education dataset (n = 4,001) (Fig. S1), the income dataset (n = 3,152) (Fig. S2), and the occupation dataset (n = 1,397) (Fig. S3).

The Maastricht study was approved by the institutional medical ethics committee (NL31329.068.10) and the Ministry of Health, Welfare and Sport of the Netherlands (permit 131088-105234-PG). All participants provided their written informed consent.

Assessment of socioeconomic position (exposure)

In this study, self-reported information on educational, income, and occupational levels was used as SEP indicators. The educational level was reported through 9 ordinal categories: 1) no education, 2) uncompleted primary education, 3) primary education, 4) lower vocational education, 5) intermediate secondary education, 6) intermediate vocational education, 7) higher secondary education, 8) higher vocational education, and 9) advanced university education. For this study, three categories were created to define the educational level: low (1 to 4), medium (5 to 7), and high (8 and 9).

Income was measured by self-reported net household income per month, and consisted of 19 categories, ranging from <€750 to >€5,000 per month. To compute equivalent income levels, household income was divided by the square root of household size. This implies that, for instance, a household of four persons has twice the needs of a single-person household.[16] Equivalent income level was categorised into low, medium, and high based on tertiles.

Participants were asked to describe their current or previous job. The job descriptions were then classified according to International Standard Classification of Occupations 2008 (ISCO-08), which is a hierarchical classification system based on education and skills required for a job.[17] The resulting codes were then converted to the International Socio-Economic Index of Occupational Status (ISEI-08),[18] which ranks occupational positions also by the average level of education and average earnings of job holders. ISEI-08 classifications were categorised as low, medium, and high occupational level based on tertiles.

Assessment of lifestyle factors (mediators)

The inclusion of lifestyle factors as potential mediators of the relationship between SEP in IHL content was based on previously reported associations between individual lifestyle factors and IHL content.[2–9]

Dietary intake was assessed using a tailor-made food frequency questionnaire (FFQ) developed using the Dutch national FFQ tool.[19] The FFQ collected information on the frequency of food consumption and the amount of consumed food and nutrients over the past 12 months. Intakes of total energy and specific nutrients was calculated using the Dutch food composition (NEVO) table 2011.[20]

Daily intake of total energy (kcal/day), alcohol (g/day), saturated fat (g/day), protein (g/day), vitamin E (mg/day), dietary fibre (g/day), fructose from fruit juice (g/day), and fructose from SSB (including sugar-containing fruit drinks and syrups) (g/day) were included as potential nutritional mediators.

Physical activity was measured using a thigh-worn accelerometer (activPAL3^{TM}, PAL Technologies, Glasgow, UK) worn for eight consecutive days.[21] Total physical activity per day was defined as the mean time spent stepping during out-of-bed time. We also calculated the amount of time per day spent on moderate-to-vigorous physical activity (MVPA) (defined as ≥100 steps/min),[22] which was used as a potential mediator effect in the present study.

Assessment of intrahepatic lipid content (outcome)

IHL content was assessed by Dixon-MRI using a 3.0 T MRI system (MAGNETOM Prismafit, Siemens Healthineers, Erlangen, Germany) with body matrix and supine radiofrequency coils.[23]

This method was validated and calibrated against proton magnetic resonance spectroscopy (^{1}H-MRS), the gold standard for non-invasive quantification of IHL, in 36 participants. After calibration, the intra-class correlation coefficient between Dixon-MRI and ^{1}H-MRS was 0.989 (95% CI: 0.979–0.994).[23] IHL content was expressed as the ratio CH_2/H_2O (× 100%).

NAFLD is defined by the presence of hepatic steatosis with an IHL content ≥5.56%[24] in the absence of excessive alcohol intake, which is defined as a daily alcohol consumption ≥30 g for men and ≥20 g for women.[25] The cut-off value for IHL content, originally expressed as CH_2 (H_2O + CH_2), corresponds to 5.89% when IHL content is expressed as CH_2/H_2O, as was done in the present study.[3]

Covariates and other measurements

All participants completed questionnaires regarding age, sex, and history of cardiovascular disease (CVD). Medication use was

assessed during medication interviews. Height, weight, waist circumference, and blood pressure were measured during a physical examination. BMI was calculated as kilogram per meter squared (kg/m^2). C-reactive protein, HbA_{1c}, and lipid profiles were measured in venous blood samples.

Participants underwent a standardised 2-h 75 g oral glucose tolerance test after fasting overnight to determine glucose metabolism status (GMS), which was defined according to the World Health Organisation 2006 criteria as normal glucose metabolism (NGM), impaired fasting glucose, and impaired glucose tolerance (combined as prediabetes status) and T2D.[26] For safety reasons, participants using insulin or with a fasting glucose level >11.0 mmol/L (determined by finger prick) did not undergo the oral glucose tolerance test. These individuals were automatically classified as having diabetes.

Insulin resistance was assessed by the homeostasis model assessment (HOMA-IR), which was calculated with the $HOMA_2$ calculator v.2.2.3 for Windows.[27]

Statistical analyses

Continuous data are presented as mean ± standard deviation (SD), or as median (IQR) in case of non-normal distribution of values. Categorical data are presented as number (%). All nutritional variables were adjusted for total energy intake by the residual method.[28] First, we studied the relationships between the SEP indicators (*i.e.* education level, income level and occupational level) (exposure) and all individual lifestyle factors (mediators) using linear regression. To fulfil the assumption of normality of linear regression, all lifestyle factors were transformed into square root values.

Second, we conducted multivariable linear regression analysis to examine the association between lifestyle factors (mediators) and IHL content (outcomes). IHL content was $\log_{10}$ transformed to fulfil the assumption of normality for linear regression. To obtain interpretable results we back-transformed the regression coefficients, which should be interpreted as the fold change (and not the additive change) in IHL content that is associated with one unit increase in certain lifestyle factor.[3]

Third, we performed multiple parallel mediation analysis (bootstrap = 10,000) to quantify to what extent the association between the SEP indicator and IHL content is mediated by each lifestyle factor. For consistence of interpretation, IHL content was also $\log_{10}$ transformed, and the total effect, direct effect and indirect effect were then back-transformed. The high SEP indicator group was used as a reference (Fig. S4). All analyses were adjusted for age, sex, and T2D, the latter because of oversampling for T2D in the Maastricht Study. Additional analyses were performed to test for the effect of interaction between SEP indicators and sex or T2D on IHL.

Several sensitivity analyses were performed. Mediation analyses were repeated after: 1) stratification by the MRI lag time (the time between basic measurements and the MRI of the liver); 2) excluding participants with T2D; 3) replacing ordinal SEP indicators by continuous SEP indicators; 4) replacement of MVPA by total physical activity; and 5) replacement of IHL content by NAFLD (binary, yes/no). Alcohol intake was excluded as a mediator in this analysis; 6) replacement of continuous mediators by binary mediators. The cut-off values for MVPA and nutritional mediators were derived from The Health Council of The Netherlands[29] and The Netherlands Nutrition Centre,[30] see Table S2.

Statistical analyses were performed using R statistical software v4.0.1 with the bruceR package[31] and SPSS v.22 (Chicago, IL, USA). A two-sided p value of <0.05 was considered statistically significant in all analyses, except for interaction tests where a less stringent significance threshold of p <0.10 was applied.

Results

Education dataset

Baseline characteristics of the overall population in the education dataset (n = 4,001) were stratified according to IHL tertiles in Table 1. Individuals included in this dataset had a somewhat better cardiometabolic profile than those who were excluded due to missing data (Table S3).

The mean age of the study population was 60 ± 9 years, 49% were female, 20% were diagnosed with T2D, 21% were diagnosed with NAFLD, and the median IHL content was 3.2% (IQR: 2.0%–6.1%). The proportion of participants with excessive alcohol intake was 11% of women and 16% of men. Compared with participants in the lowest IHL tertile, those in the highest were older and more often male, had a lower educational level and less MVPA. Intake of total energy, alcohol, saturated fat, and fructose from SSB were higher, while intake of dietary fibre were lower in the highest IHL tertile when compared with individuals in the lowest IHL tertile.

Participants in the highest IHL tertile were metabolically more unhealthy than participants in the lowest IHL tertile, as suggested by the lower HDL cholesterol, and higher BMI, serum triglycerides, HbA1c, HOMA-IR, c-creative protein, systolic and diastolic blood pressure, and prevalence of prediabetes, T2D and NAFLD. Moreover, the prevalence of CVD and the use of medication (including lipid-modifying and antihypertensive medication) were higher in the highest IHL tertile.

Age, sex, and T2D adjusted analyses revealed that both individuals with low and medium education levels were characterised by a 5.6%- and 2.6%-fold higher IHL content, independent of the underlying aetiology, compared with individuals with higher education levels (beta: 1.056, 95% CI: 1.030–1.083; and beta: 1.026, 95% CI: 1.001–1.052, respectively). These associations were stronger in women than in men (p for interaction: <0.001 and 0.060, respectively; Table S4).

Individuals with lower education had less MVPA, a higher intake of fructose from SSB, and lower intake of alcohol, vitamin E, dietary fibre, and fructose from fruit juice, than individuals with high education (Table 2). Similar associations were found for medium education (compared with high education), albeit with a weaker strength of association (Table 2).

Table 3 shows the association between lifestyle factors and IHL content after adjustment for age, sex, and T2D. A higher intake of alcohol, protein, fructose from SSB and fruit juice were associated with a higher IHL content, whereas inverse associations were observed for dietary fibre and MVPA.

Mediation analyses showed that MVPA, dietary fibre intake and fructose intake from SSB were statistically significant mediators of the association between lower education (*vs.* higher education) and IHL content, with a proportion-mediated effect of 8.9%, 5.5%, and 6.3%, respectively (Fig. 1A and Table S5). In contrast, alcohol intake was a suppressor of this association, with a proportion-mediated effect of -23.0% (Fig. 1A and Table S5). Comparable results were found for the association between medium education (*vs.* higher education) (Table S5). Of note, a

Table 1. Characteristics of the study population (education dataset), stratified according to intrahepatic lipid (IHL) content (N = 4,001).

Characteristics	Total (N = 4,001)	First tertile (n = 1,334)	Second tertile (n = 1,333)	Third tertile (n = 1,334)
Intrahepatic lipid content, %	3.2 (2.0–6.1)	1.7 (1.3–2.0)	3.2 (2.7–3.9)	8.3 (6.1–12.6)
Age, yr	60 ± 9	57 ± 9	60 ± 8	61 ± 8
Women, %	49	62	46	40
Education, % low/medium/high	32/28/40	26/30/44	32/27/41	38/27/35
Moderate to vigorous physical activity, min/day	51.4 (36.6–69.6)	56.1 (40.6–73.6)	52.7 (38.5–72.1)	45.1 (31.9–62.1)
Total energy intake, kcal/day	2,077 (1,722–2,487)	2,028 (1,699–2,449)	2,088 (1,749–2,518)	2,104 (1,707–2,500)
Alcohol intake, g/day	8.6 (1.8–18.8)	7.8 (1.5–15.7)	9.6 (2.5–19.6)	8.2 (1.6–20.9)
Participant with excessive alcohol intake (female/male), %	11/16	9/10	12/15	12/20
Saturated fat intake, g/day	27.3 (20.5–35.4)	26.3 (20.2–34.3)	27.6 (20.5–35.7)	28.0 (20.8–36.1)
Protein intake, g/day	82.0 (69.0–96.9)	80.8 (68.0–94.9)	82.8 (69.5–98.1)	82.5 (69.4–97.6)
Vitamin E intake, mg/day	12.5 (9.7–16.0)	12.5 (9.8–16.0)	12.5 (9.8–16.2)	12.5 (9.6–15.8)
Dietary fibre intake, g/day	26.1 (21.3–31.8)	26.4 (21.4–32.0)	26.6 (21.7–32.2)	25.4 (21.1–30.9)
Fructose intake from SSB, g/day	0.4 (0.0–2.8)	0.3 (0.0–2.1)	0.3 (0.0–2.4)	0.6 (0.0–3.8)
Fructose intake from fruit juice, g/day	0.9 (0.1–3.8)	1.0 (0.2–3.8)	0.9 (0.1–3.8)	0.9 (0.1–3.9)
BMI, kg/m^2	26.5 ± 4.1	24.3 ± 3.0	26.3 ± 3.6	28.9 ± 4.1
Waist circumference, cm	93.8 ± 12.6	85.6 ± 9.7	93.7 ± 11.0	101.9 ± 11.4
Office SBP, mmHg	133 ± 17	128 ± 17	133 ± 17	138 ± 16
Office DBP, mmHg	76 ± 10	73 ± 10	76 ± 9	78 ± 9
Antihypertensive medication, %	33	20	32	48
Total cholesterol, mmol/L	5.3 ± 1.1	5.3 ± 1.0	5.3 ± 1.1	5.2 ± 1.2
HDL cholesterol, mmol/L	1.6 ± 0.5	1.8 ± 0.5	1.6 ± 0.5	1.4 ± 0.4
LDL cholesterol, mmol/L	1.7 ± 0.6	1.7 ± 0.5	1.7 ± 0.6	1.5 ± 0.6
Triglycerides, mmol/L	1.2 (0.9–1.7)	1.0 (0.8–1.2)	1.3 (0.9–1.6)	1.7 (1.1–2.1)
Lipid-modifying medication, %	28	17	28	39
HbA1c, %	5.5 (5.3–5.9)	5.4 (5.1–5.6)	5.4 (5.3–5.8)	5.7 (5.4–6.5)
HbA1c, mmol/mol	37 (34–41)	35 (32–38)	36 (34–40)	39 (35–47)
HOMA-IR	1.34 (0.95–2.01)	1.03 (0.77–1.34)	1.28 (0.93–1.77)	1.90 (1.31–2.73)
GMS, % (NGM/prediabetes/type 2 diabetes/other types of diabetes)	64/15/20/1	82/9/8/1	71/13/15/1	41/21/38/0
C-reactive protein, μg/ml	1.2 (0.6–2.5)	0.8 (0.5–1.7)	1.1 (0.6–2.4)	1.7 (0.9–3.4)
History of CVD, %	13	10	13	16
NAFLD, %	21	0	0	64

Data are reported as mean ± standard deviation, median (IQR), or n (%) as appropriate. Nutritional variables represent absolute intake values. BMI, body mass index; CVD, cardiovascular disease; DBP, diastolic blood pressure; GMS, glucose metabolism status; HbA1c, glycated haemoglobin A1c; HOMA-IR, homeostasis model assessment-insulin resistance; IHL, intrahepatic lipid; NAFLD, non-alcoholic fatty liver disease; NGM, normal glucose metabolism; SBP, systolic blood pressure; SSB, sugar-sweetened beverages.

Table 2. Association between education (exposure) and lifestyle factors (mediators) (N = 4,001).

	Medium *vs.* high education		Low *vs.* high education	
Lifestyle factors	Coefficient (95% CI)	*p* value	Coefficient (95% CI)	*p* value
Moderate to vigorous physical activity, min/day	-0.136 (-0.266 to 0.006)	0.041	-0.249 (-0.378 to -0.120)	<0.001
Total energy intake, kcal/day	0.097 (-0.362 to 0.555)	0.680	0.092 (-0.363 to 0.547)	0.693
Alcohol intake, g/day	-0.435 (-0.565 to 0.304)	<0.001	-0.679 (-0.809 to -0.55)	<0.001
Saturated fat intake, g/day	0.015 (-0.032 to 0.062)	0.535	0.002 (-0.045 to 0.048)	0.948
Protein intake, g/day	-0.006 (-0.058 to 0.046)	0.825	-0.029 (-0.080 to 0.023)	0.271
Vitamin E intake, mg/day	-0.013 (-0.046 to 0.021)	0.464	-0.049 (-0.082 to 0.016)	0.004
Dietary fibre intake, g/day	-0.027 (-0.068 to 0.014)	0.202	-0.074 (-0.115 to 0.033)	<0.001
Fructose intake from SSB, g/day	0.177 (0.097 to 0.257)	<0.001	0.272 (0.193 to 0.352)	<0.001
Fructose intake from fruit juice, g/day	-0.090 (-0.165 to 0.015)	0.019	-0.111 (-0.186 to 0.037)	0.004

Regression coefficients should be interpreted as the (square root) difference in lifestyle factor between individuals with medium or low education level compared with individuals with a higher education level (reference group). Results are based on linear regression adjusted for age, sex, and type 2 diabetes. A two-sided *p* value of <0.05 was considered statistically significant. All nutritional variables were energy-adjusted by the residual method. SSB, sugar-sweetened beverage.

substantial proportion the relationship between education and IHL content was not explained by these lifestyle factors (direct effect>total effect; Fig. 1A and Table S5).

When the analyses were stratified by sex and MRI lag time (*i.e.* the time between the basic measurements and the MRI), the suppressive effect of alcohol was less pronounced in women (Table S6) and participants with more than 1 year MRI lag time (Table S7). When the analysis was performed in participants without T2D (n = 3,184), similar results were observed (Table S8).

Replacement of IHL content by NAFLD (n = 4,001) showed that individuals with low and medium education were characterised by a 44.3% and 44.8 % higher risk of NAFLD than individuals with a higher education (OR: 1.443, 95% CI: 1.179–1.766 and OR: 1.448, 95%CI: 1.179–1.777, respectively). Mediation analyses demonstrated similar trends, although dietary fibre intake was no longer statistically significant (Fig. 1B and Table S9). Replacement of educational level (as an ordinal trait) by education on a continuous scale did not materially alter the results (Table S10),

Table 3. Multivariable-adjusted associations of lifestyle factors (mediator) and IHL content (outcome) (N = 4,001).

Lifestyle factors	Coefficient (95% CI)	*p* value
Moderate to vigorous physical activity, min/day	0.999 (0.998–0.999)	<0.001
Total energy intake, kcal/day	1.000 (1.000–1.000)	0.623
Alcohol intake, g/day	1.003 (1.002–1.004)	<0.001
Saturated fat intake, g/day	1.002 (1.000–1.004)	0.062
Protein intake, g/day	1.002 (1.001–1.002)	0.001
Vitamin E intake, mg/day	0.999 (0.996–1.002)	0.627
Dietary fibre intake, g/day	0.996 (0.994–0.999)	0.002
Fructose intake from SSB, g/day	1.003 (1.001–1.006)	0.013
Fructose intake from fruit juice, g/day	1.003 (1.000–1.006)	0.026

Regression coefficients should be interpreted as the fold change in IHL content that is associated with one unit increase in lifestyle factor. All nutritional variables were energy-adjusted by the residual method. Results are based on linear regression adjusted for age, sex, and type 2 diabetes. A two-sided *p* value of <0.05 was considered statistically significant. IHL, intrahepatic lipid; SSB, sugar-sweetened beverages.

nor did replacement of MVPA by total physical activity (data not shown). Finally, replacement of continuous mediators by binary mediators showed that a large proportion of the total effect of education on IHL content was not mediated by traditional lifestyle factors (Table S11).

Income dataset

Baseline characteristics of the participants in the income dataset (n = 3,152) are shown in Table S12. Both individuals with low and medium income were characterised by a higher, albeit non-significant, IHL content compared with individuals with higher income (beta: 1.018, 95%CI: 0.990–1.048 and beta: 1.023, 95%CI: 0.995–1.052, respectively). These associations were again stronger in women than in men (p for interaction: 0.126 and 0.059, respectively; Table S4).

The associations between income level with lifestyle factors and lifestyle factors with IHL content are presented in Supplementary Tables 13 and 14, and were comparable to the results for educational level.

MVPA and intake of fructose from SSB were statistically significant mediators of the association between low income (*vs.* high income) and IHL content, with a proportion-mediated effect of 37.9% and 29.8%, respectively (Table S15). Intake of alcohol and protein were suppressors of this association, with a proportion-mediated effect of -13.1% and -90.8%, respectively (Table S15). Stratification by sex and MRI lag time again showed that the suppressive effect of alcohol was less pronounced in women (Table S16) and participants with ≥1 year MRI lag time (Table S17). Analysis in non-T2D participants showed generally similar results (Table S18).

Replacement of the IHL content by NAFLD (n = 3,152) showed that individuals with low and medium income were characterised by a 52.8% and 44.2% higher risk of NAFLD compared with individuals with higher income (OR: 1.528, 95% CI: 1.203–1.941 and OR: 1.442, 95% CI: 1.137–1.829, respectively). The mediators were essentially similar (Table S19). Replacement of income level (as an ordinal trait) by income on a continuous scale (Table S20) and MVPA by total physical activity did not affect the outcomes (data not shown). Table S21 showed the results when the continuous mediators were replaced by binary mediators.

Occupation dataset

Baseline characteristics of the participants in the occupation dataset (n = 1,397) are shown in Table S22. Both individuals with low and medium occupational level were characterised by a higher IHL content compared with individuals with a high occupational level (beta: 1.053, 95% CI: 1.008–1.100 and beta: 1.039, 95%CI: 0.995–1.084, respectively). These associations were also stronger in women than in men (*p* for interaction: 0.002 and 0.201, respectively; Table S4).

The associations of occupational level with lifestyle factors and lifestyle factors with IHL content are presented in Tables S23 and S24, and were generally comparable to the results stratified by education level.

Alcohol intake was a statistically significant suppressor of the association between low occupational levels (*vs.* high occupational level) and IHL content, with a proportion-mediated effect of -25.1% (Table S25). After stratification according to sex, MRI lag time yielded similar outcomes (Tables S26 and 27). Analysis in participants without T2D showed similar results (Table S28).

Replacement of IHL content by NAFLD (n = 1,397) showed that individuals with low and medium occupational levels were characterised by a 47.5% and 36.0% higher risk of NAFLD compared with individuals with a higher occupational level (OR: 1.475, 95% CI: 1.039–2.092 and OR: 1.360, 95%CI: 0.958–1.932, respectively). Mediation analyses showed similar results (Table S29). Replacement of occupational level (as an ordinal trait) by ISEI-08 (Table S30) and MVPA by physical activity did not affect the outcomes (data not shown). Table S31 shows the analysis results for binary mediators.

Discussion

In this study, we found that individuals with low and medium SEP had a higher IHL content, independent of aetiology, and had a greater risk of NAFLD compared with individuals with high SEP. These associations were stronger in women than in men. MVPA, and dietary intake of fibre and fructose from SSB were significant mediators of the association between low education (*vs.* high education) and IHL content, whereas alcohol intake was a statistically significant suppressor.

To date, only one study has addressed the relationships between SEP, dietary intake, physical activity and NAFLD at the population level. That study reported that high education, but not household income was associated with a reduced risk of NAFLD. This association was partially mediated by high quality diet and increased physical activity.[32] Our results are in agreement and further extend these findings. First, we were able to include occupational status as another SEP indicator, which showed generally similar results. The absence of statistical significance for some mediators (in comparison to the analyses conducted for educational level) is at least in part explained by a lack of statistical power due to the smaller dataset. Second, we studied specific food items that are known to affect IHL

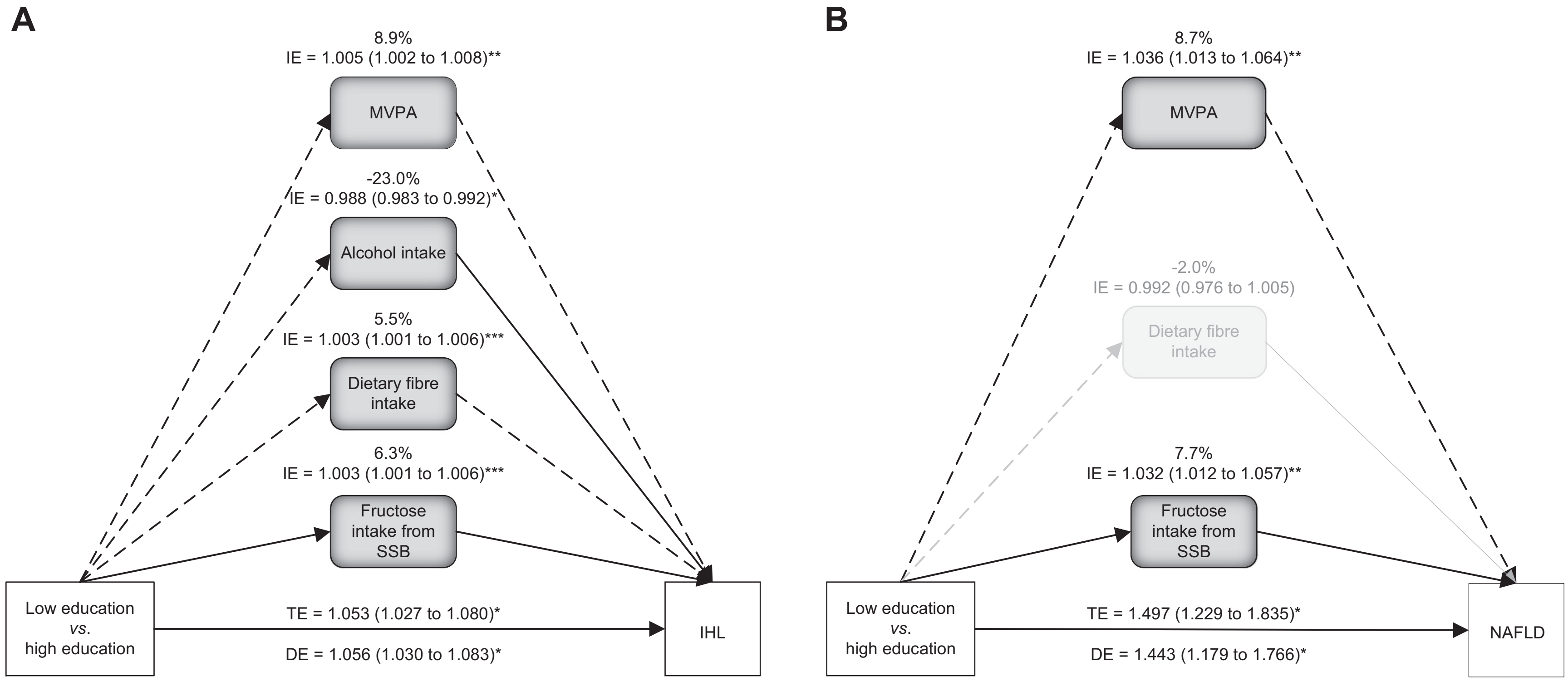

Fig. 1. Mediators of the association between education (low *vs.* high) and IHL content. Association independent of (A) aetiology, and (B) NAFLD. Only statistically significant mediators of the association between education and IHL content are shown. Dietary fibre intake is no longer statistically significant in the model for NAFLD (presented in grey). Alcohol intake is excluded from the mediators in the model for NAFLD. The results for all mediators included in the models are presented in Tables S5 and S9. Results are based on parallel multiple mediation analysis (bootstrap = 10,000) adjusted for age, sex, and type 2 diabetes. *p <0.05; **p <0.01; ***p <0.001. A two-sided p value of <0.05 is considered statistically significant. All nutritional variables are energy-adjusted by the residual method. Solid arrows indicate positive relationships, whereas dashed arrows indicate inverse relationships. Total, direct, and indirect effects are expressed as fold change in IHL content (A) or as odds ratios of NAFLD (B). Proportion of mediation = Log_{10}(indirect effect)/$\log_{10}$(total effect). DE, direct effect; IE, indirect effect; IHL, intrahepatic lipid; MVPA, moderate to vigorous physical activity; NAFLD, non-alcoholic fatty liver disease; SSB, sugar-sweetened beverage; TE, total effect.

content,[2,3,5–8] which may aid targeted policy strategies, as will be described below. Lastly, we used IHL content, independent of aetiology, as the main outcome, since a high intake of alcohol and a metabolically unhealthy lifestyle can co-exist in the same individual in real-life.[10] We found that alcohol played an important mediating role in explaining the association between low SEP and IHL content. This mediation was suppressive since alcohol intake was higher in individuals with a high SEP, which has also been observed in other studies.[33–35] Of note, the suppressive effect of alcohol was less pronounced in women, which may explain why all associations between the SEP indicators and IHL content were stronger in women. Moreover, the suppressive effect of alcohol was much stronger for the association of low income (*vs.* high income) and IHL content, which may explain the non-significant relationship between income and IHL content. We subsequently narrowed our analysis by focusing on NAFLD as the secondary outcome, which is solely attributable to metabolic risk abnormalities.[25]

The interpretation of the current findings is complex, as educational level does not reflect health literacy alone, as income level is not the only indicator of financial resources. For instance, studies have shown that both adults and children correctly rate SSBs as the most unhealthy beverages.[36] Furthermore, alternative healthy beverages are available at similar or even lower costs. The mediation effect of fructose from SSB on the relationship of both educational and income level with IHL content is, therefore, most likely explained by other related SEP factors. For example, people living in socioeconomic deprived areas have greater access to fast food and takeaway food outlets.[37,38] Conversely, financial resources may affect physical activity levels, since free access to leisure facilities has led to increased physical activity particularly in individuals with low SEP.[39,40]

The implications of this study are severalfold. First, it illustrates the complex pathogenesis of NAFLD. The global prevalence of NAFLD has risen to epidemic proportions[41] and, therefore, cannot be treated by new drugs or lifestyle interventions at the individual level alone. Instead, preventive measures should be undertaken at the societal level, including improvement of health literacy, introduction of (dis)incentives (*e.g.* subsidised physical activity and levies on SSBs, which have been shown to be effective[42]), and re-designing of the living environment (*e.g.* enhancement of neighbourhood walkability[39] and reduced accessibility to fast food outlets, particularly near schools), in order to stimulate a healthy lifestyle and, consequently, prevent NAFLD.

Second, and equally important, our study convincingly showed that a substantial degree of the relationship between SEP and IHL content was not explained by the traditional lifestyle factors. We found that the direct effect, *i.e.* the relationship between SEP and IHL not accounted for by the mediators under investigation, was even greater than the total effect. This may be attributed to the fact that the net mediating effect of traditional lifestyle factors (= indirect effect) was negative due to the substantial suppressive effects of alcohol. This implicates the existence of other, yet unmeasured mediators. Future studies are warranted to unveil the role of other putative mediators, including exposure to endocrine disruptors (such as phthalate) and air pollution, which both have been associated with both low SEP and NAFLD,[43–48] as these may involve other preventive measures.

The study has several strengths and limitations. First, we used data from a large population-based cohort that was extensively phenotyped using state-of-the-art methods. This allowed for an accurate estimation of IHL content. Second, by using both IHL content, a continuous trait, and NAFLD, a binary trait, in our primary and secondary analyses, respectively, we show that our results are relevant and robust. Third, as mentioned before, instead of performing simple causal mediation analyses, we applied multiple parallel mediation analysis, which can evaluate a host of indirect effects by multiple mediators, revealing the complex mechanisms between SEP, lifestyle factors, and IHL content by decomposing the total effects. Indirect effects of different mediators reflect the importance of different pathways and, therefore, provide useful information for tailored policy making. Nonetheless, our study also has specific limitations. First, SEP and dietary intake were based on self-reported information and, hence, can be prone to bias. Moreover, although the FFQ has been validated against 24-h dietary recalls for intakes of mono- and disaccharides, fruit and drinks, it has not been validated for SSB.[19] Second, we only considered the amount of alcohol intake thereby ignoring any drinking patterns, which may be associated with both exposure and outcome. Third, MRI measurements were introduced to the extensive set of measurements at a later point in time, which resulted in an attenuation of the mediation effects without affecting the proportion not mediated by the traditional lifestyle factors. Fourth, we included lifestyle factors that have been associated with IHL[2–9] as potential mediators. It can, therefore, not be excluded that other, less traditional lifestyle factors can be used to explain the relationship between SEP and IHL content/NAFLD. Finally, a substantial number of participants were excluded from the analyses, mainly because MRI measurements were not implemented in all participants. This led to the inclusion of participants who had a somewhat more beneficial cardiometabolic profile.

In conclusion, this population-based study demonstrates that MVPA, alcohol consumption, dietary fibre, and fructose intake from SSB are mediators/suppressors of the association between low SEP and IHL accumulation, independent of aetiology. These findings suggest that societal measures to alleviate the global burden of NAFLD are warranted. Furthermore, our observations also suggest that lifestyle improvement in patients with low SEP will not normalise their risk, since a substantial proportion of SEP factors, IHL content, and NAFLD incidence could not be associated with traditional lifestyle mediators. Further studies are needed to identify other mediators that may explain the relationship between SEP and IHL content.

Abbreviations

CVD, cardiovascular disease; FFQ, food frequency questionnaire; GMS, glucose metabolism status; HOMA, homeostasis model assessment; IHL, intrahepatic lipid; IR, insulin resistance; ISCO, International Standard Classification of Occupations; ISEI, International Socio-Economic Index; MRI, magnetic resonance imaging; NAFLD, non-alcoholic fatty liver disease; NGM, normal glucose metabolism; SEP, socioeconomic position; SSB, sugar-sweetened beverage.

Financial support

ZR was supported by Chinese Scholarship Council. The Maastricht Study was supported by the European Regional Development Fund via OP-Zuid, the Province of Limburg, the Dutch Ministry of Economic Affairs (grant 31O.041), Stichting De Weijerhorst (Maastricht, the Netherlands), the Pearl String Initiative Diabetes (Amsterdam, the Netherlands), School for Cardiovascular Diseases (CARIM, Maastricht, the Netherlands), Care and Public Health Research Institute (CAPHRI; Maastricht, the Netherlands), School for Nutrition and Translational Research in Metabolism (NUTRIM; Maastricht, the Netherlands), Stichting Annadal (Maastricht, the Netherlands), Health Foundation Limburg (Maastricht, the Netherlands), and by unrestricted grants from JanssenCilag B.V. (Tilburg, the Netherlands), Novo Nordisk Farma B.V. (Alphen aan den Rijn, the Netherlands), Sanofi-Aventis Netherlands B.V. (Gouda, the Netherlands), and Medtronic (Tolochenaz, Switzerland).

Conflicts of interest

All authors declare no conflicts of interest related to this manuscript.

Please refer to the accompanying ICMJE disclosure forms for further details.

Authors' contributions

Concept and design of the study: ZR, MCGJB, HB, and CDAS. Generation, collection, assembly, analysis and/or interpretation of data and drafting the manuscript: ZR and MCGJB. All authors contributed to the intellectual content of the manuscript and review of the manuscript and approved the final version for submission. MCGJB is the guarantor of this work and, as such, had full access to all the data in the study and takes responsibility for the integrity of the data and the accuracy of the data analysis.

Data availability statement

Data are available from The Maastricht Study for any researcher who meets the criteria for access to confidential data; the corresponding author may be contacted to request data.

Acknowledgements

We thank all the participants in The Maastricht Study and contributions from all the co-authors.

Supplementary data

Supplementary data to this article can be found online at https://doi.org/10.1016/j.jhepr.2023.100855.

References

[1] Riazi K, Azhari H, Charette JH, Underwood FE, King JA, Afshar EE, et al. The prevalence and incidence of NAFLD worldwide: a systematic review and meta-analysis. Lancet Gastroenterol Hepatol 2022;7:851–861.
[2] Mouzaki M, Allard JP. The role of nutrients in the development, progression, and treatment of nonalcoholic fatty liver disease. J Clin Gastroenterol 2012;46:457–467.
[3] Buziau AM, Eussen S, Kooi ME, van der Kallen CJH, van Dongen MCJM, et al. Fructose intake from fruit juice and sugar-sweetened beverages is associated with higher intrahepatic lipid content: the Maastricht Study. Diabetes Care 2022;45:1116–1123.
[4] Ter Horst KW, Serlie MJ. Fructose consumption, lipogenesis, and non-alcoholic fatty liver disease. Nutrients 2017;9:981.
[5] Luukkonen PK, Sädevirta S, Zhou Y, Kayser B, Ali A, Ahonen L, et al. Saturated fat is more metabolically harmful for the human liver than unsaturated fat or simple sugars. Diabetes Care 2018;41:1732–1739.
[6] Markova M, Pivovarova O, Hornemann S, et al. Isocaloric diets high in animal or plant protein reduce liver fat and inflammation in individuals with type 2 diabetes. Gastroenterology 2017;152:571–585.e8.
[7] Rietman A, Sluik D, Feskens EJM, Kok FJ, Mensink M. Associations between dietary factors and markers of NAFLD in a general Dutch adult population. Eur J Clin Nutr 2018;72:117–123.
[8] Perumpail BJ, Li AA, John N, Sallam S, Shah ND, Kwong W, et al. The role of vitamin E in the treatment of NAFLD. Diseases 2018;6:86.
[9] Keating SE, Hackett DA, George J, Johnson NA. Exercise and non-alcoholic fatty liver disease: a systematic review and meta-analysis. J Hepatol 2012;57:157–166.
[10] Buyco DG, Martin J, Jeon S, Hooks R, Lin C, Carr R. Experimental models of metabolic and alcoholic fatty liver disease. World J Gastroenterol 2021;27:1–18.
[11] Dieteren C, Bonfrer I. Socioeconomic inequalities in lifestyle risk factors across low- and middle-income countries. BMC Public Health 2021; 21:951.
[12] Kabir A, Miah S, Islam A. Factors influencing eating behavior and dietary intake among resident students in a public university in Bangladesh: a qualitative study. PLoS One 2018;13:e0198801.
[13] Vega-Salas MJ, Caro P, Johnson L, Armstrong MEG, Papadaki A. Socio-economic inequalities in physical activity and sedentary behaviour among the Chilean Population: a systematic review of observational studies. Int J Environ Res Public Health 2021;18:9722.
[14] Lee H, Cashin AG, Lamb SE, Hopewell S, Vansteelandt S, VanderWeele TJ, et al. A guideline for reporting mediation analyses of randomized trials and observational studies: the AGReMA Statement. JAMA 2021;326: 1045–1056.
[15] Schram MT, Sep SJ, van der Kallen CJ, Dagnelie PC, Koster A, Schaper N, et al. The Maastricht Study: an extensive phenotyping study on determinants of type 2 diabetes, its complications and its comorbidities. Eur J Epidemiol 2014;29:439–451.
[16] Organization for Economic Co-operation and Development. Quality review of the OECD database on household incomes and poverty and the OECD Earnings Database; Part I. Paris, France: OECD; 2012.
[17] International Standard Classification of Occupations. International classification of occupations 2008-structure, group definitions and correspondence tables. 1st ed. Genova, Switzerland: ILO; 2012.
[18] Ganzeboom HBG. A new international socio-economic index [ISEI] of occupational status for the International Standard Classification of Occupation. In: Annual conference of international social survey programme; 2010. Lisbon, Portugal.
[19] van Dongen MC, Wijckmans-Duysens NEG, den Biggelaar LJ, Ocké MC, Meijboom S, Brants HA, et al. The Maastricht FFQ: development and validation of a comprehensive food frequency questionnaire for the Maastricht study. Nutrition 2019;62:39–46.
[20] NEVO online of Dutch food compostition tablevol. 2022. The Netherlands: RIVM/Voedingscentrum; 2011, 2011/version3.0. https://www.rivm.nl/en/dutch-food-composition-database. [Accessed 6 September 2023].
[21] van der Berg JD, Willems PJ, van der Velde JH, Savelberg HH, Schaper NC, Schram MT, et al. Identifying waking time in 24-h accelerometry data in adults using an automated algorithm. J Sports Sci 2016;34:1867–1873.
[22] Tudor-Locke C, Aguiar EJ, Han H, Ducharme SW, Schuna Jr JM, Barreira TV, et al. Walking cadence (steps/min) and intensity in 21-40 year olds: CADENCE-adults. Int J Behav Nutr Phys Act 2019;16:8.
[23] Brouwers M, Simons N, Kooi ME, de Ritter R, van Dongen MCJM, Eussen SJPM, et al. Intrahepatic lipid content is independently associated with soluble E-selectin levels: the Maastricht study. Dig Liver Dis 2022;54:1038–1043.
[24] Szczepaniak LS, Nurenberg P, Leonard D, Browning JD, Reingold JS, Grundy S, et al. Magnetic resonance spectroscopy to measure hepatic triglyceride content: prevalence of hepatic steatosis in the general population. Am J Physiol Endocrinol Metab 2005;288:E462–E468.
[25] European association for the study of the liver (EASL); European association for the study of diabetes (EASD); European association for the study of obesity (EASO). EASL-EASD-EASO clinical practice guidelines for the management of non-alcoholic fatty liver disease. Diabetologia 2016;59: 1121–1140.
[26] World Health Organization & International Diabetes Federation. Definition and diagnosis of diabetes mellitus and intermediate hyperglycaemia: report of a WHO/IDF consultation World Health Organization. 2006. Geneva. http://https://apps.who.int/iris/handle/10665/43588. [Accessed 6 September 2023].
[27] The Oxford Centre for Diabetes EaMDTU. HOMA2 calculator. https://www.dtu.ox.ac.uk/homacalculator/. (Accessed 25 August 2023).
[28] Willett W, Stampfer MJ. Total energy intake: implications for epidemiologic analyses. Am J Epidemiol 1986;124:17–27.
[29] Weggemans RM, Backx FJG, Borghouts L, Chinapaw M, Hopman MTE, Koster A, et al. The 2017 Dutch physical activity guidelines. Int J Behav Nutr Phys Act 2018;15:58.

[30] The Netherlands Nutrition Centre. Nutrition Center Encyclopedia. https://www.voedingscentrum.nl/nl/encyclopedie. (Accessed 06 September 2023).
[31] Bao H. bruceR: Broadly useful convenient and efficient R Functions. 2022; https://psychbruce.github.io/bruceR/. [Accessed 6 September 2023].
[32] Vilar-Gomez E, Nephew LD, Vuppalanchi R, et al. High-quality diet, physical activity, and college education are associated with low risk of NAFLD among the US population. Hepatology 2022;75:1491–1506.
[33] Li J, Wu B, Tevik K, Krokstad S, Helvik AS. Factors associated with elevated consumption of alcohol in older adults-comparison between China and Norway: the CLHLS and the HUNT Study. BMJ Open 2019;9:e028646.
[34] Molander RC, Yonker JA, Krahn DD. Age-related changes in drinking patterns from mid- to older age: results from the Wisconsin longitudinal study. Alcohol Clin Exp Res 2010;34:1182–1192.
[35] Skourlis N, Massara P, Patsis I, Peppa E, Katsouyanni K, Trichopoulou A. Long-term trends (1994–2011) and predictors of total alcohol and alcoholic beverages consumption: the EPIC Greece Cohort. Nutrients 2021;13:3077.
[36] Bucher T, Siegrist M. Children's and parents' health perception of different soft drinks. Br J Nutr 2015;113:526–535.
[37] Althoff T, Nilforoshan H, Hua J, Leskovec J. Large-scale diet tracking data reveal disparate associations between food environment and diet. Nat Commun 2022;13:267.
[38] Larson NI, Story MT, Nelson MC. Neighborhood environments: disparities in access to healthy foods in the U.S. Am J Prev Med 2009;36:74–81.
[39] Chalikavada R, Broder JC, O'Hara RL, Xue W, Gasevic D. The association between neighbourhood walkability and after-school physical activity in Australian schoolchildren. Health Promot J Austr 2021;32:182–188.
[40] Zhang R, Zhang CQ, Lai PC, Cheng W, Schüz B, Kwan MP. Park environment and moderate-to-vigorous physical activity in parks among adolescents in a high-density city: the moderating role of neighbourhood income. Int J Health Geogr 2021;20:35.
[41] Younossi ZM, Koenig AB, Abdelatif D, Fazel Y, Henry L, Wymer M. Global epidemiology of nonalcoholic fatty liver disease-Meta-analytic assessment of prevalence, incidence, and outcomes. Hepatology 2016;64:73–84.
[42] Andreyeva T, Marple K, Marinello S, Moore TE, Powell LM. Outcomes following taxation of sugar-sweetened beverages: a systematic review and meta-analysis. JAMA Netw Open 2022;5:e2215276.
[43] Foulds CE, Treviño LS, York B, Walker CL. Endocrine-disrupting chemicals and fatty liver disease. Nat Rev Endocrinol 2017;13:445–457.
[44] Rui W, Guan L, Zhang F, Zhang W, Ding W. PM2.5-induced oxidative stress increases adhesion molecules expression in human endothelial cells through the ERK/AKT/NF-κB-dependent pathway. J Appl Toxicol 2016;36:48–59.
[45] Corsini E, Budello S, Marabini L, Galbiati V, Piazzalunga A, Barbieri P, et al. Comparison of wood smoke PM2.5 obtained from the combustion of FIR and beech pellets on inflammation and DNA damage in A549 and THP-1 human cell lines. Arch Toxicol 2013;87:2187–2199.
[46] Folkmann JK, Risom L, Hansen CS, Loft S, Møller P. Oxidatively damaged DNA and inflammation in the liver of dyslipidemic ApoE-/- mice exposed to diesel exhaust particles. Toxicology 2007;237:134–144.
[47] Gray SC, Edwards SE, Miranda ML. Race, socioeconomic status, and air pollution exposure in North Carolina. Environ Res 2013;126:152–158.
[48] Montazeri P, Thomsen C, Casas M, de Bont J, Haug LS, Maitre L, et al. Socioeconomic position and exposure to multiple environmental chemical contaminants in six European mother-child cohorts. Int J Hyg Environ Health 2019;222:864–872.

Journal of Hepatology, Volume *5*

Supplemental information

Traditional lifestyle factors partly mediate the association of socioeconomic position with intrahepatic lipid content: The Maastricht study

Zhewen Ren, Hans Bosma, Anke Wesselius, Simone J.P.M. Eussen, M. Eline Kooi, Carla J.H. van der Kallen, Annemarie Koster, Marleen M.J. van Greevenbroek, Pieter Dagnelie, Coen D.A. Stehouwer, and Martijn C.G.J. Brouwers

Traditional lifestyle factors partly mediate the association of socioeconomic position with intrahepatic lipid content: The Maastricht study

Zhewen Ren, Hans Bosma, Anke Wesselius, Simone JPM Eussen, M. Eline Kooi, Carla J.H. van der Kallen, Annemarie Koster, Marleen van Greevenbroek, Pieter Dagnelie, Coen DA Stehouwer, Martijn CGJ Brouwers

Table of contents

Supplementary figures...2

Supplementary tables..separate excel file

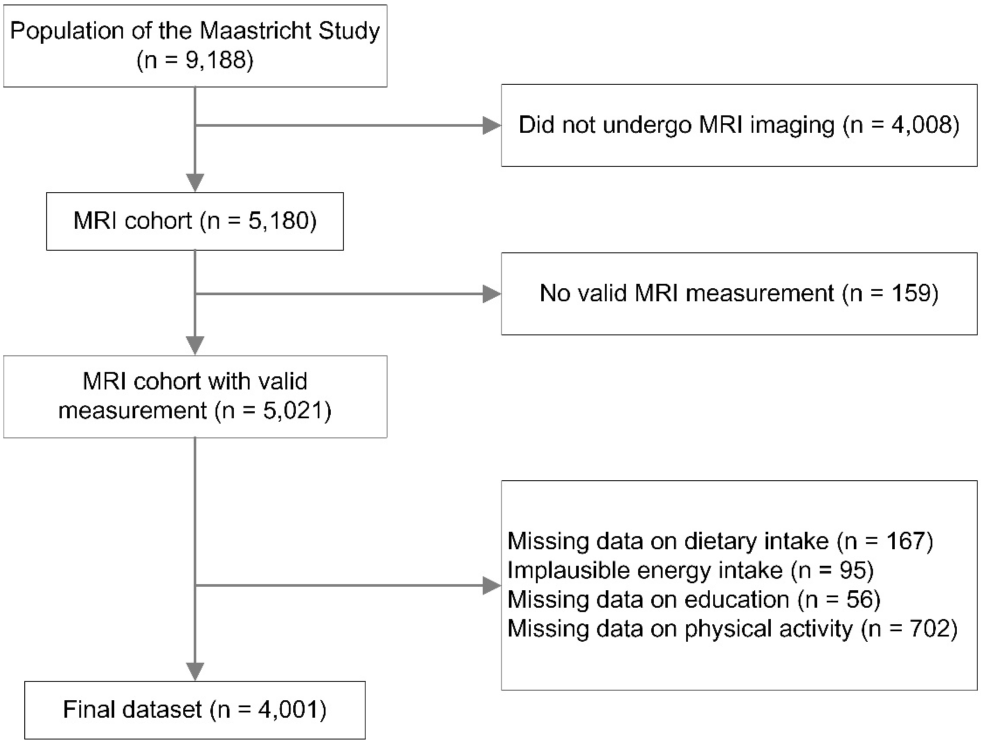

Fig. S1. Flowchart of the education dataset.

Abbreviation: MRI: Magnetic resonance imaging.

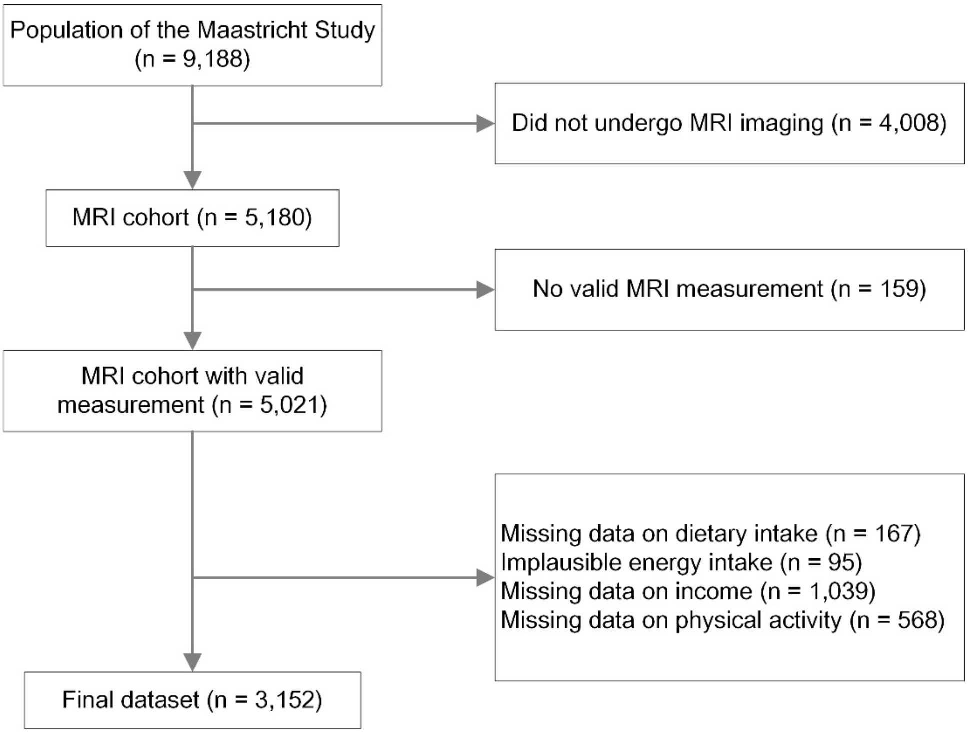

Fig. S2. Flowchart of the income dataset.

Abbreviation: MRI: Magnetic resonance imaging.

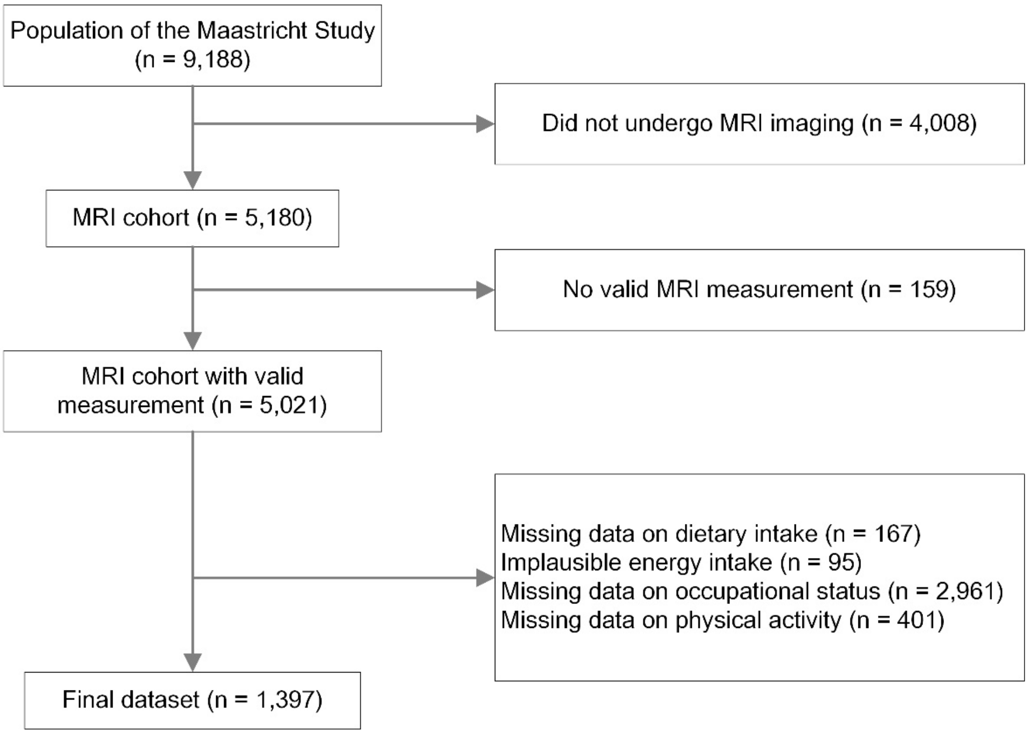

Fig. S3. Flowchart of the occupation dataset.

Abbreviation: MRI: Magnetic resonance imaging.

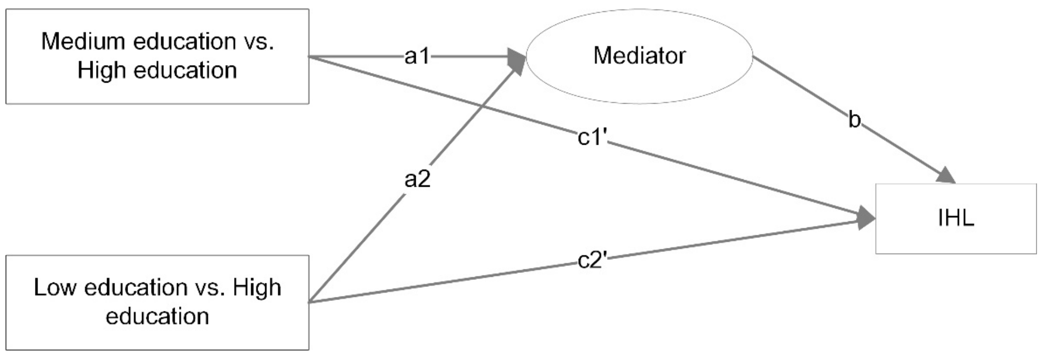

Fig. S4. **Multiple parallel mediation model to assess the mediation effects of lifestyle factors on the relationship between education and IHL content.**

a1*b and a2*b represent the indirect effects, whereas c1', c2' represent the direct effects. Abbreviations: IHL: intrahepatic lipid content.
